# Predicting severe COVID-19 in the Emergency Department

**DOI:** 10.1016/j.resplu.2020.100042

**Published:** 2020-10-21

**Authors:** Aleksander Rygh Holten, Kristin Grotle Nore, Caroline Emilie Van Woensel Kooy Tveiten, Theresa Mariero Olasveengen, Kristian Tonby

**Affiliations:** a Department of Acute Medicine, Oslo University Hospital, Oslo, Norway; b Institute of Clinical Medicine, University of Oslo, Oslo, Norway; c Department of Infectious Diseases, Oslo University Hospital, Oslo, Norway; d Division of Emergencies and Critical Care, Oslo University Hospital, Oslo, Norway

**Keywords:** COVID- 19, National Early Warning Score2 (NEWS2), qSOFA, Systemic Inflammatory Response Syndrome (SIRS), CURB-65, Pneumonia Severity Index (PSI)

## Abstract

**Background:**

COVID-19 may lead to severe disease, requiring intensive care treatment and challenging the capacity of health care systems. The aim of this study was to compare the ability of commonly used scoring systems for sepsis and pneumonia to predict severe COVID-19 in the emergency department.

**Methods:**

Prospective, observational, single centre study in a secondary/tertiary care hospital in Oslo, Norway. Patients were assessed upon hospital admission using the following scoring systems; quick Sequential Failure Assessment (qSOFA), Systemic Inflammatory Response Syndrome criteria (SIRS), National Early Warning Score 2 (NEWS2), CURB-65 and Pneumonia Severity index (PSI). The ratio of arterial oxygen tension to inspiratory oxygen fraction (P/F-ratio) was also calculated. The area under the receiver operating characteristics curve (AUROC) for each scoring system was calculated, along with sensitivity and specificity for the most commonly used cut-offs. Severe disease was defined as death or treatment in ICU within 14 days.

**Results:**

38 of 175 study participants developed severe disease, 13 (7%) died and 29 (17%) had a stay at an intensive care unit (ICU). NEWS2 displayed an AUROC of 0.80 (95% confidence interval 0.72−0.88), CURB-65 0.75 (0.65−0.84), PSI 0.75 (0.65−0.84), SIRS 0.70 (0.61–0.80) and qSOFA 0.70 (0.61−0.79). NEWS2 was significantly better than SIRS and qSOFA in predicating severe disease, and with a cut-off of5 points, had a sensitivity and specificity of 82% and 60%, respectively.

**Conclusion:**

NEWS2 predicted severe COVID-19 disease more accurately than SIRS and qSOFA, but not significantly better than CURB65 and PSI. NEWS2 may be a useful screening tool in evaluating COVID-19 patients during hospital admission.

**Trial registration:**

: ClinicalTrials.gov Identifier: NCT04345536. (https://clinicaltrials.gov/ct2/show/NCT04345536).

## Introduction

COVID-19 was first discovered in Wuhan, China, in December 2019^1^ and has since spread to every continent. The pandemic caused by SARS-CoV-2 has had a devastating impact on healthcare worldwide,^2^ exceeding local health-care capacity in many regions of the world.^3^^,^^4^ It is estimated that about 5% of COVID-19 patients develop critical disease.^5^ Viral pneumonia is the most common organ manifestation, and many patients need respiratory support.^6^^,^^7^ Although the majority of patients with COVID-19 admitted to hospital can be managed on general wards with supplemental oxygen, there is limited knowledge on how to identify patients that will ultimately need invasive ventilatory support. A reliable screening tool to identify patients at risk would help allocate limited monitoring resources.

Several tools have been developed for risk stratification in patients with *sepsis* and *pneumonia*. *Sepsis*^8^, ^9^, ^10^, ^11^ and *pneumonia*^12^, ^13^, ^14^ scoring systems have been evaluated separately in COVID-19, but few comprehensive, prospective comparison of these tools in COVID-19 have been published.^15^ The accuracy of sepsis and pneumonia scoring systems in COVID-19 is therefore still uncertain.^16^

Commonly applied scoring systems for *sepsis* in the emergency department (ED) include; a) the Quick Sequential Failure Assessment (qSOFA) score ^17^ b) Systemic Inflammatory Response Syndrome criteria (SIRS),^18^ and c) National Early Warning Score 2 (NEWS2).^19^ For pneumonia, the two most commonly used scoring systems are CURB-65^20^^,^^21^ and Pneumonia Severity Index (PSI).^22^ Both have been prospectively validated to predict mortality. PSI is far more comprehensive and time consuming than CURB-65, though currently recommended by the American Thoracic Society and Infectious Diseases Society of America.^23^ Lastly, the ratio between arterial oxygen tension and inspiratory oxygen fraction (P/F-ratio) is used in the assessment of Acute Respiratory Distress Syndrome (ARDS), defined as P/F-ratio ≤300 mmHg (40 kPa).^24^ Although not strictly a scoring system, the P/F-ratio is considered to be important in assessing respiratory failure in critically ill patients,^25^ and may be another tool for the initial assessment.

In this prospective observational study, we have evaluated and compared the predictive characteristics of commonly used scoring systems for sepsis and pneumonia applied to a cohort of consecutive COVID-19 patients admitted to our hospital. Our aim was to evaluate their accuracy in the ED in predicting the development of severe COVID-19 infection within 14 days after hospital admittance, in order to assist initial triage and allocation of limited monitoring resources.

**Quick Sequential Organ Failure Assessment (qSOFA) (1 point each; score range, 0–3 points)**^17^

Altered mental state

Systolic blood pressure ≤100 mmHg

Respiratory rate ≥22 breaths/min

**Systemic Inflammatory Response Syndrome (SIRS) (1 point each; score range, 0–4 points)**^18^

Temperature >38 °C or <36 °C

Heart rate > 90 beats/min

Respiratory rate >20 breaths/min or PaCO2 < 4.3 kPa

White blood cell count >12 000 cells/mm^3^ or < 4000 cells/mm^3^

**CURB-65 (1 point each; score range, 0–5 points)** ^20^

Confusion

Urea > 7 mmol/l

Respiratory rate ≥30 breaths/min

Systolic blood pressure <90 mmHg or diastolic blood pressure ≤ 60 mmHg

Age ≥ 65 years

**Pneumonia severity index (risk class I–V)**^22^

*Step 1*. If any, assign patient to *risk class II-V* according to step 2. If non, assign patient to *risk class I*

Age > 50 years

History of neoplastic disease, congestive heart failure, cerebrovascular disease, renal disease or liver disease

Any of the following abnormalities on physical examination: Altered mental status, pulse ≥ 125 beats/min, respiratory rate ≥ 30 breaths/min, systolic blood pressure <90 mmHg or temperature <35 °C or ≥40 °C.

*Step 2*. Point scoring system (point assigned for each characteristic). If the score is ≤70 the patient is assigned to risk class II, 71–90 to risk class III, 91–130 to risk class IV and >130 risk to risk class V.

Age (1 point/year)

Female sex (10 points)

Nursing home resident (10 points)

Neoplastic illnesses (30 points)

Liver disease (20 points)

Congestive heart failure (10 points)

Cerebrovascular disease (10 points)

Renal failure (10 points)

Altered mental state (20 points)

Respiratory rate ≥30 breaths/min (20 points)

Systolic blood pressure <90 mmHg (20 points)

Temperature <35 °C or ≥40 °C (15 points)

Pulse ≥ 125 beats/min (10 points)

Arterial pH < 7.35 (30 points)

Blood urea nitrogen ≥ 11 mmol/l (20 points)

Sodium <130 mmol/l (20 points)

Glucose >14 mmol/l (10 points)

Haematocrit < 30% or Haemoglobin <9 g/dl (10 points)

PaO2 < 60 mmHg (>8 kPa) (10 points)

Pleural effusion (10 points)

**National Early Warning Score 2 (NEWS2) (score range 0–20)**^19^

The following six physiological parameters are scored from 0 to 3.

Respiratory Rate

Oxygen Saturations (with lower oxygen saturation targets for patients with confirmed hypercapnia)

Temperature

Systolic blood pressure

Heart Rate

Level of consciousness

Additional 2 point if any Supplemental Oxygen is administered.

## Methods

All SARS-CoV2-positive patients >18 years old, admitted to the OUH during the period from 01.03.20 to 30.06.20 were evaluated for study participation. Patients transferred from other hospitals, and patients electively hospitalized for medical conditions unrelated to SARS-CoV-2, were excluded. Since the aim of this study was to evaluate the value of the scoring systems in the ED, only the calculation of qSOFA, SIRS-criteria, CURB-65, PSI, NEWS2 and P/F-ratio from the patient’s first assessment at the hospital were included. Severe disease was defined as death or treatment in ICU within 14 days after admittance. There were no established criteria for ICU admission. The decision to transfer a patient to ICU was taken by experienced intensivist on duty.

The included patients were included in the quality registry “Covid19 OUS”, at Oslo University Hospital (OUH). The quality registry was approved by the data protection officer at OUH on March 13th 2020 (Reference number 20/08822). Informed consent was waived in accordance with the data protection officer. Patients were included prospectively, but before March 13th, due to the acute onset of the pandemic in Norway, the first eight patients were included retrospectively. The register contains all patients hospitalized with confirmed SARS-CoV-2 (positive reverse transcriptase polymerase chain reaction (RT-PCR)) regardless of symptoms and clinical findings. Identification of COVID- 19 positive patients admitted to OUH has been done by cross-checking results of positive SARS-CoV-2 RT- PCR provided by the Department of Microbiology at OUH, and by use of the diagnose coding system “International Classification of Diseases” (ICD10) by identifying the diagnosis codes J12.8 (other viral pneumonia) in combination with U07.1 (COVID-19 identified). The clinicians who assessed the patients were otherwise unrelated to the study. Data were extracted from patient journals and recorded electronically by register staff in the Medinsight registration tool. The records were controlled by two of the authors (ARH, TMO).

This paper has been developed according to the STARD-guidelines for reporting diagnostic accuracy studies.^26^

### Statistical analysis

The area under the receiver operating characteristics curve (AUROC) for severe disease was calculated for each scoring system. Sensitivity, specificity, positive and negative predicative values of the most commonly used cut-off values for SIRS-criteria (2/4), qSOFA (2/3), CURB-65 (2/5), PSI (3/5) and NEWS (5), together with a P/F-ratio less than 300 mmHg (40 K Pa), were determined. Patients with missing data were excluded from the individual calculation.

Continuous variables are given in median and interquartile ranges, and compared using the independent Student’s *t* test. Categorical variables are given in numbers and percentages, and were compared with the chi-square test. Statistical analyses were calculated using the IBM SPSS Statistics (version 26) and GraphPad Prism (version 8). 95% confidence intervals for sensitivity, specificity and positive and negative predicative values were calculated by Medcalc (https://www.medcalc.org/). P-values less than 0.05 were considered significant.

## Results

### Patient characteristics

**Fig. 1:**
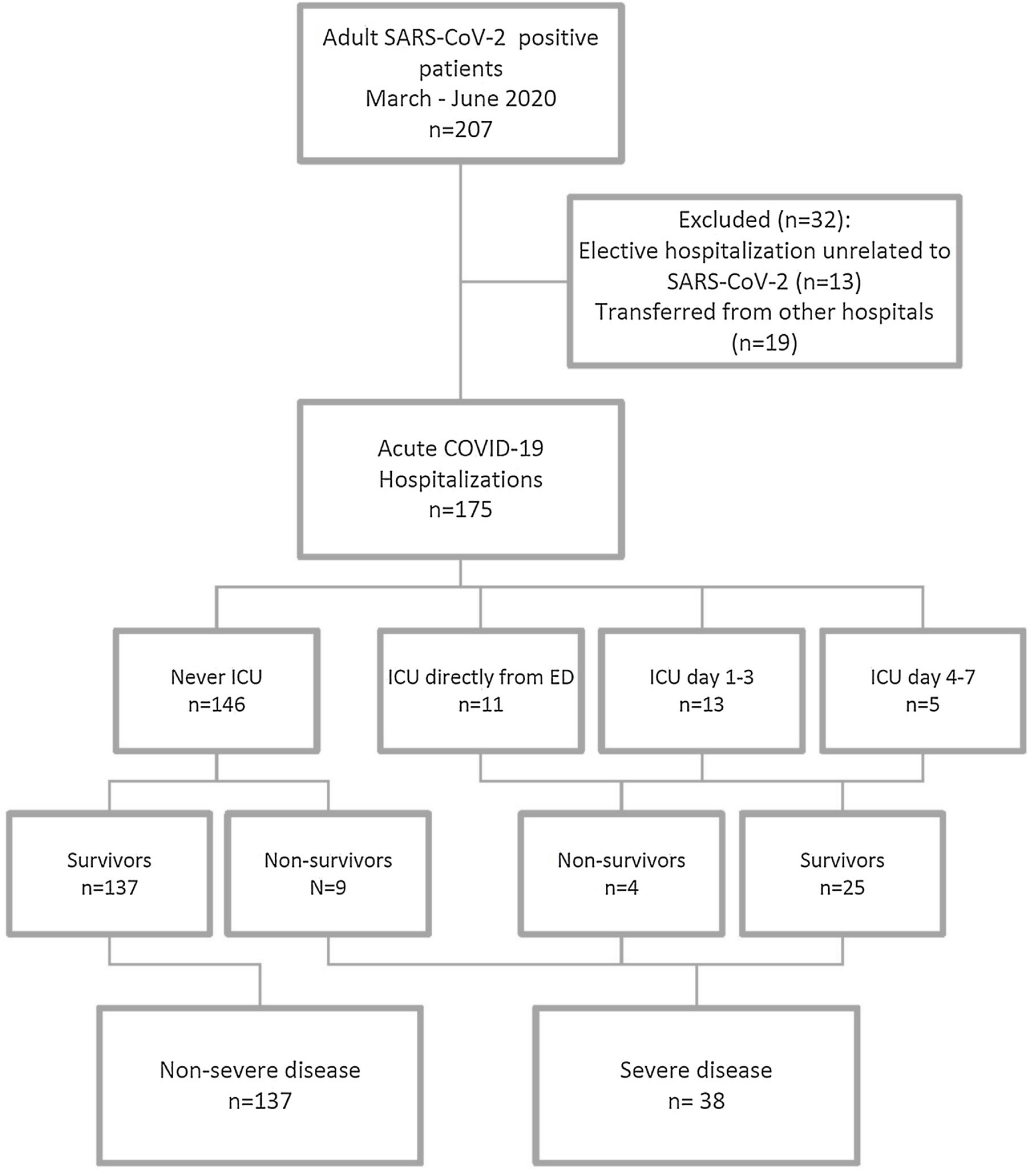
Flow chart over study participants.

A total of 207 adult patients with confirmed COVID-19 were admitted to the hospital from March to June 30th. 19 patients transferred from other hospitals and 13 patients electively admitted for nonCOVID related conditions were excluded (Fig. 1), leaving 175 for further analysis. 169 patients had sufficient data from the first assessment to calculate qSOFA, SIRS, CURB-65, PSI and NEWS2. P/F-ratio could be calculated in 136 patients. Within the first two weeks after admittance, 13 patients (7%) died and 29 (17%) were transferred to one of four ICUs treating patients with COVID-19. 38 patients had severe disease according to the definition of the study protocol. All ICU-patients were admitted to the ICU within 7 days and 83% within the first 3 days. The surge capacity of the hospital was never exceeded. 21 of 29 (72%) ICU- patients were mechanically ventilated.

The patients who developed severe disease were older, had more comorbidity, frailty and limitation of treatment. Baseline characteristics, see Table 1.

**Table 1:** Baseline characteristics.

Variable	All (n = 175)	Severe disease (n = 38)	Non-severe disease (n = 137)	p-value (severe VS non-severe)
Age, median (IQR), y	59 (26)	65 (27)	54 (25)	0.002
Male sex, No (%)	102 (58%)	27 (71%)	75 (55%)	0.071
Limitation of treatment, No (%)	32 (18%)	13 (34%)	19 (14%)	0.004
Charlson Comorbidity Index, median (IQR)	1 (2)	1 (4)	0 (2)	0.001
Clinical Frailty Index, median (IQR)	3 (1)	3 (2)	2 (1)	0.001
Duration of symptoms before admittance, median (IQR), days	8 (7)	7 (5)	8 (7)	0.40

	All (n = 169)	Severe disease (n = 38)	Non-severe disease (n = 131)	p-value (severe VS non-severe)
qSOFA ≥ 2, No (%)	17 (10%)	10 (26%)	7 (5%)	<0.001
SIRS ≥ 2, No (%)	93 (55%)	30 (79%)	63 (48%)	0.001
CURB-65 ≥ 2, No (%)	49 (29%)	22 (58%)	27 (21%)	<0.001
PSI ≥ 3, No (%)	66 (39%)	27 (71%)	39 (30%)	<0.001
NEWS ≥ 5, No (%)	83 (49%)	31 (82%)	52 (40%)	<0.001
NEWS ≥ 6, No (%)	66 (39%)	29 (76%)	37 (28%)	<0.001
NEWS ≥ 7, No (%)	47 (28%)	25 (66%)	22 (17%)	<0.001

	All (n = 136)	Severe disease (n = 37)	Non-severe disease (n = 99)	p-value (severe VS non-severe)
PaO_2_/FiO_2_ ≤ 300 mmHg (≤40 kPa), No (%)	53 (39%)	26 (70%)	27 (27%)	<0.001

### Main outcome

**Fig. 2:**
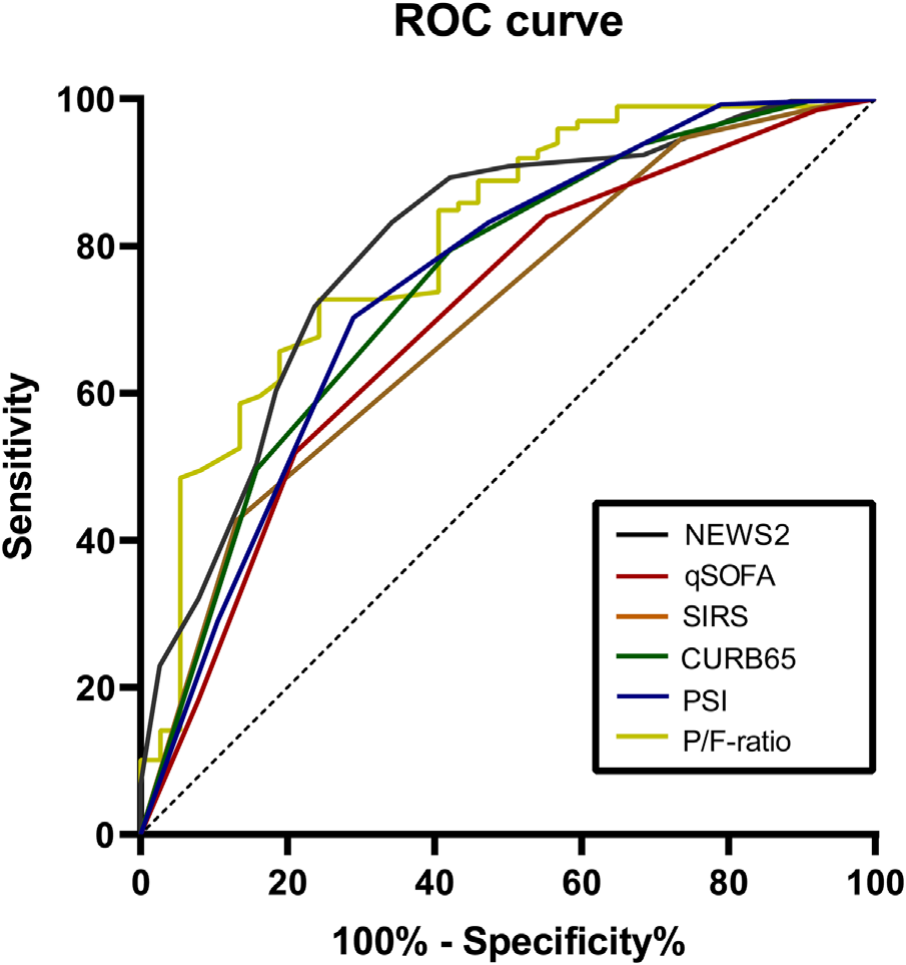
Receiver operating characteristics (ROC) curve for predicting severe COVID-19. The curves for NEWS2, qSOFA, SIRS, CURB65 and PSI are based on 169 patients (38 severe, and 131 non-severe), whereas the curve for P/F-ratio are based 136 patients (37 severe and 99 non-severe).

NEWS2 displayed an AUROC of 0.80 (95% confidence interval 0.71−0.88), CURB-65 0.76 (0.67−0.84), PSI 0.74 (0.64−0.84), SIRS 0.70 (0.61–0.80) and qSOFA 0.70 (0.62−0.78). NEWS2 were significantly better than qSOFA and SIRS (Fig. 2 and Table 2), and comparable with CURB-65 and PSI. NEWS2 cut-off set to five points demonstrated a sensitivity of 82% and a specificity of 60% in detecting severe COVID-19 (Table 3). qSOFA had a sensitivity of 26% and a specificity of 95%. SIRS displayed a sensitivity of 79% and a specificity of 52%. The pneumonia prediction scores CURB-65 and PSI had sensitivities of 58% and 71% and specificities of 80% and 70%, respectively. The positive predicative values (PPV) of the scoring systems were generally low. NEWS2 ≥ 5 showed the highest negative predicative value (NPV) of 92%, only significantly higher than qSOFA ≥ 2.

**Table 2:** Area under the receiver operating characteristics curve (AUROC) for the scoring system predicting severe COVID-19 (defined as death or treatment at ICU). The AUROC for P/F-ratio is based on 37 and 99 patients with severe and non- severe disease respectively. The AUROC for the other variables are based on 38 patients with severe disease and 131 patients with non-severe disease.

Test Result Variables	AUROC	95% Confidence Interval		p-value VS AUROC for NEWS2
		Lower Bound	Upper Bound	
NEWS2	0,80	0.72	0.88	n/a
qSOFA	0.70	0.61	0.79	<0.001
SIRS	0.70	0.61	0.80	0.015
CURB-65	0.75	0.65	0.84	0.33
PSI	0.75	0.65	0.84	0.36
P/F-ratio	0.81	0.72	0.89	0.48

**Table 3:** Sensitivity, specificity, positive predicative value (PPV) and negative predicative value (NPV) for the most commonly used cut-off values for the scoring systems. 95% confidence intervals in brackets. NEWS2 is showed with three different cut off values.

	Sensitivity % (95% CI)	Specificity % (95% CI)	PPV % (95% CI)	NPV% (95% CI)
qSOFA ≥ 2	26 (13–43)	95 (89–98)	59 (37–78)	82 (78–84)
SIRS ≥ 2	79 (63–90)	52 (43–61)	32 (27–38)	90 (82–94)
CURB-65 ≥ 2	58 (41–74)	80 (71–86)	45 (35–56)	87 (82–91)
PSI ≥ 3	71 (54–85)	70 (62–78)	41 (33–49)	89 (83–93)
NEWS2 ≥ 5	82 (66–92)	60 (51–68)	37 (32–43)	92 (85–96)
NEWS2 ≥ 6	76 (60–89)	72 (63–79)	44 (36–52)	91 (85–94)
NEWS2 ≥ 7	66 (49–80)	83 (76–89)	53 (42–64)	89 (84–93)
PaO_2_/FiO_2_ ≤ 300 mmHg (≤40 kPa)	70 (53–84)	73 (63–81)	49 (40–59)	87 (80–92)

The AUROC for P/F-ratio for predicting severe disease was 0.81, which is comparable with NEWS2, CURB-65 and PSI. The sensitivity and specificity for the P/F-ratio with cut-off ≤300 mmHg (≤40 kPa) were 70% and 73%, respectively.

## Discussion

This prospective observational study of five different scoring systems applied on COVID-19 patients at admittance to hospital found that NEWS2 predicts severe disease in COVID-19 patients significantly better than the sepsis scoring systems qSOFA and SIRS. The AUROC was not significantly higher for NEWS2 than for CURB-65 and PSI.

With a cut-off of NEWS2 set to five, the sensitivity of 82% and the NPV of 92% are moderately good. However, the specificity of 60% and PPV of only 37% makes it challenging to use in clinical practice. When the NEWS2 cut-off value is increased, both the specificity and the PPV increase, but at the expense of sensitivity. NEWS2 is currently used in assessing patients with suspected severe infections in several EDs around the world.^27^^,^^28^ Even though a single assessment during hospital admission obviously has limited predictive capability, NEWS2 might still be useful as a clinical decision tool also in COVID-19. Reliable identification of patients at high risk of developing severe COVID-19 could have important implications. A predictive tool would be helpful in selecting the patients most likely to benefit from additional monitoring, inclusion in interventional trials or receiving intensive care.

There have been several attempts at developing new tools to predict development of severe COVID-19.^29^ Liang and colleagues developed a prognostic scoring system consisting of ten factors including X-ray abnormalities, age, symptoms, comorbid conditions and biomarkers.^30^ The score is validated in four additional cohorts and obtained an AUROC of 0.88 both in the development cohort and the validation cohorts. It is even launched as a freely available online risk calculator. While promising, the score is demanding in use and requires several diagnostic tests before the score can be calculated.

In contrast to sepsis, which commonly presents with multiorgan failure, COVID-19 is often characterized by solitary respiratory failure.^7^^,^^31^ In contrast to the other sepsis scoring tools, three of seven parameters in the NEWS2 score relate to degree of respiratory failure, and this could explain its relatively high performance compared to the other scoring systems we evaluated. However, maximal scores in NEWS2 are reached at respiratory rate ≥ 25, oxygen saturation ≤ 91% and the need for any supplemental oxygen – parameters often far exceeded in COVID-19 patients at time of admission. It is possible that the prognostic accuracy of the NEWS2 score could be improved by modifying the score. For instance, respiratory rate ≥ 40 breaths per minute, oxygen saturation ≤ 80% and need for ≥ 10 L of oxygen might further add to the total score. Novel predictive strategies, whether based on new artificial Intelligence technology^32^ or modifications of existing scores, need to be prospectively tested in adequately sized training and validation cohorts.

Of the 175 hospitalized COVID-19 patients included in our study, 38 were defined as severe disease; non-survivors or by need of intensive care treatment. Thus, adequate clinical observations are of outmost importance. This is hindered by a multitude of infection control measures such as time-consuming donning and doffing of personal protective equipment. It is therefore important that the routines for observations are simple and well documented. Serial assessments using NEWS2 is a reasonable practical approach, and may possibly identify deteriorating patients. While NEWS2 did not perform significantly better than CURB65 and PSI, it is simple to use, include only easily accessible clinical parameters, while CURB-65 and PSI both include laboratory analyses. P/F-ratio assessment require blood gas analysis, and may therefore be difficult to perform outside of the ICU.

It must be emphasized that COVID-19 is mainly a pulmonary disease. We suggest that the NEWS2 score in COVID-19 patients is supplemented with monitoring the degree of oxygen requirement and more detailed grading of respiratory rate and signs of patient exhaustion, as well as the P/F- ratio if feasible.

This study has some limitations. It is a single centre study which may limit the generalizability, and the relatively small number of participants limits the certainty of our analysis. The study was conducted in the early phase of the pandemic, and there may have been changes in clinical practice and routines during the study period. However, we do not think this affected data collection. Notable strengths are that all participants are well characterized in a large prospective quality register that comprise a complete consecutive patient set, including the first COVID-19 patients admitted to our hospital.

In conclusion, our study revealed that NEWS2 was equivalent to CURB65, PSI and P/F-ratio, but more accurate than SIRS and qSOFA, in predicting severe disease among patients hospitalized for COVID-19. However, the value of single assessments is limited, and hospitalized patients must be adequately monitored for signs of deterioration.

## Funding

The study was funded by Oslo University Hospital. It did not receive any external funding.

## Registration

The study was registered as an observational study in ClinicalTrials.gov. The ClinicalTrials.gov Identifier is NCT04345536.

## Conflicts of interest

Dr Olasveengen has received research grants from Zoll Foundation and Laerdal Foundation. No other disclosures were reported.

## Ethics approval

Informed consent was waived in accordance with the data protection officer (case number 20/07119) due to the status as a quality register with reporting of aggregated patient data with no risk of identification of personal sensitive information.

## Consent for publication

The publication was approved by data protection officer at Oslo University Hospital (case number 20/08822, April 04, 2020).

## Availability of data and material (data transparency)

Due to the nature of this research, with data from a quality register with waived consent, data is not available do to ethical and legal restrictions.

## Code availability

Not applicable.

## CRediT authorship contribution statement

**Aleksander Rygh Holten:** Conceptualization, Formal analysis, Writing - original draft, Writing - review & editing, Supervision. **Kristin Grotle Nore:** Formal analysis, Investigation, Writing - review & editing. **Caroline Emilie Van Woensel Kooy Tveiten:** Formal analysis, Investigation, Writing - review & editing. **Theresa Mariero Olasveengen:** Methodology, Formal analysis, Investigation, Writing - review & editing, Resources. **Kristian Tonby:** Methodology, Formal analysis, Investigation, Writing - review & editing, Resources, Supervision.
